# Ultrasound-guided percutaneous drainage of infected pancreatic necrosis

**DOI:** 10.1007/s00464-013-2831-9

**Published:** 2013-02-13

**Authors:** Marek Wroński, Włodzimierz Cebulski, Dominika Karkocha, Maciej Słodkowski, Łukasz Wysocki, Mieczysław Jankowski, Ireneusz W. Krasnodębski

**Affiliations:** Department of General, Gastroenterological and Oncological Surgery, Medical University of Warsaw, ul. Banacha 1A, 02-097 Warsaw, Poland

**Keywords:** Acute pancreatitis, Infected necrosis, Pancreatic necrosectomy, Pancreatic necrosis, Percutaneous catheter drainage

## Abstract

**Background:**

The role of percutaneous drainage in the management of infected pancreatic necrosis remains controversial, and ultrasound-guided technique is rarely used for this indication. The purpose of this study was to evaluate the safety and efficacy of sonographically guided percutaneous catheter drainage for infected pancreatic necrosis.

**Methods:**

The patient group consisted of 16 men and 2 women. The mean age of the patients was 47 years. The median computed tomography severity index of acute pancreatitis was 10 points. Percutaneous catheter drainage was performed under sonographic guidance using preferably retroperitoneal approach, and transperitoneal access in selected cases. The medical records and imaging scans were reviewed retrospectively for each patient.

**Results:**

Percutaneous catheter drainage resulted in a complete resolution of infected pancreatic necrosis in 6 of 18 patients (33 %). Twelve of 18 patients who were initially managed with PCD required eventually necrosectomy (67 %). The most common reason for crossover to surgical intervention was persistent sepsis (*n* = 7). Open necrosectomy was performed in 4 of these patients, and 3 patients underwent successful minimally invasive retroperitoneal necrosectomy. Five patients required conversion to open surgery because of procedure-related complications. In 3 cases, there was leakage of the necrotic material into the peritoneal cavity. Two other patients experienced hemorrhagic complications. Overall mortality rate was 17 %. The size of the largest necrotic collection in patients who were successfully treated with percutaneous drainage decreased by a median of 76 % shortly after the procedure, whereas it decreased only by a median of 16 % in cases of failure of percutaneous drainage.

**Conclusions:**

Ultrasound-guided percutaneous catheter drainage used in infected pancreatic necrosis is a technique with acceptably low morbidity and mortality that may be the definitive treatment or a bridge management to necrosectomy. A negligible decrease in size of the necrotic collection predicts failure of percutaneous drainage.

Pancreatic and peripancreatic necrosis occurs in 10–20 % of acute pancreatitis [1]. In 40–70 % of necrotizing pancreatitis, necrosis becomes infected [2]. Traditionally, infected pancreatic necrosis (IPN) has been an indication for open surgical debridement. Recently, several techniques of minimally invasive debridement of pancreatic necrosis have been introduced. Percutaneous catheter drainage (PCD) was the first minimal access technique used for the treatment of infected pancreatic necrosis. Although this technique is highly successful in intra-abdominal abscesses or infected pancreatic pseudocysts, its role in the management of infected pancreatic necrosis remains controversial. Percutaneous catheter drainage used for this indication has often been criticized for its poor ability to remove the solid debris. Percutaneous drainage is usually performed under the guidance of computed tomography (CT), whereas sonographically controlled PCD has rarely been reported [3, 4]. So far there are no reliable criteria to predict which patients might benefit from percutaneous drainage.

The purpose of this study was to evaluate the safety and efficacy of ultrasound-guided percutaneous catheter drainage in patients with infected pancreatic necrosis. We also attempted to define the factors that could predict in which patients PCD might prove successful. Ultrasound-guided percutaneous catheter drainage was introduced to practice in our institution in 2007, and the study reports our preliminary experience with this technique.

## Materials and methods

### Patients

Between January 2007 and December 2011, 262 patients were admitted to our department with the diagnosis of acute pancreatitis. Seventy six patients (29 %) had necrotizing pancreatitis. Thirty-three patients (43 %) developed infection of pancreatic necrosis. Ultrasound-guided percutaneous catheter drainage was performed as an initial intervention in 18 of these 33 patients (54 %). The remaining 14 patients (42 %) underwent primary surgical necrosectomy, and one patient died before the operation could be performed. In 6 of these 14 patients, the treating surgeon preferred open surgical debridement. In other 6 patients, percutaneous access to the necrosis was regarded technically impossible due to bowel interposition or abundant gas within the necrotic collection that precluded a safe placement of the catheter. In one patient, the main indication for surgical intervention was a concomitant toxic megacolon secondary to severe Clostridium difficile infection. In another patient, open surgical debridement was indicated because of active bleeding into the collection. The study group included only the patients with pancreatic and/or peripancreatic necrosis in whom infection was confirmed by a positive culture of a specimen taken at the time of the first drainage procedure or there was peripancreatic gas on preoperative CT scans. Patients with sterile pancreatic necrosis, pseudocysts or abscesses were excluded from the study. Patients with sterile pancreatic necrosis that became infected after percutaneous drainage and patients referred to our department after having PCD done elsewhere were not included in this study either. The medical records and imaging scans were reviewed retrospectively for each patient. A decrease in size of the necrotic collections was evaluated based on CT scans. The size of the necrotic collections was calculated as the surface area of an ellipse using the dimensions of the largest collection. The change in size of the necrotic collection was estimated only in patients who had a follow-up CT within 4 weeks after the initial PCD. There were only 3 patients meeting these criteria in the group successfully treated by PCD alone and also 3 patients in the group who failed percutaneous drainage.

The study group consisted of 16 men and 2 women. The mean ± standard deviation age of the patients was 47 ± 14 years. The etiology of acute pancreatitis was most commonly alcohol abuse. The median computed tomography severity index of acute pancreatitis (CTSI) was 10 points (range, 4–10 points). Three of 18 patients had only extrapancreatic necrosis. In the remaining patients, necrosis involved both the pancreas and peripancreatic tissues. Computed tomography revealed acute necrotic collection in eight patients and walled-off necrosis in ten patients. The demographic and clinical characteristics of the patients are summarized in Table 1.

**Table 1:** Demographic and clinical characteristics of the patients

Characteristics	Successful PCD (*n* = 6)	Failed PCD (*n* = 12)
Age, years, median (range)	38.5 (30–57)	45 (32–75)
Sex, M/F	5:1	11:1
Etiology		
Alcohol	5	8
Stones	1	2
Hypertriglyceridemia	0	1
Idiopathic	0	1
CTSI, points, median (range)	9 (4–10)	10 (4–10)
>50 % necrosis, *n* (%)	3 (50)	6 (50)
Extrapancreatic necrosis alone, *n* (%)	1 (17)	2 (17)
ANC/WON	1:5	7:5
Necrosis extending down to the lower pole of the kidney/limited to the lesser sac	5:1	10:2
Patients with single/multiple organ dysfunction before PCD, *n* (%)	0 (0)	2 (17)
CRP, mg/L, median (range)	278.5 (181–501)	251 (122–492)
WBC, ×10^9^/L, median (range)	16.2 (7.6–23.8)	13.4 (5.6–45.0)
Single/mixed flora	4:2	8:4

*PCD* percutaneous catheter drainage, *CTSI* computed tomography severity index of acute pancreatitis, *ANC* acute necrotic collection, *WON* walled-off necrosis, *CRP* C-reactive protein, *WBC* white blood cell count

### Therapeutic management

All the patients with acute pancreatitis received initially conservative treatment including intravenous fluids, nutritional support and prophylactic antibiotics in selected cases. Radiological or surgical intervention was postponed as long as possible to allow maximal demarcation and liquefaction of the devitalized pancreatic and peripancreatic tissues. Fine needle aspiration (FNA) for recognition of pancreatic infection is not practiced routinely in our institution and the necessity of intervention was based on clinical, radiological and laboratory grounds.

**Fig. 1:**
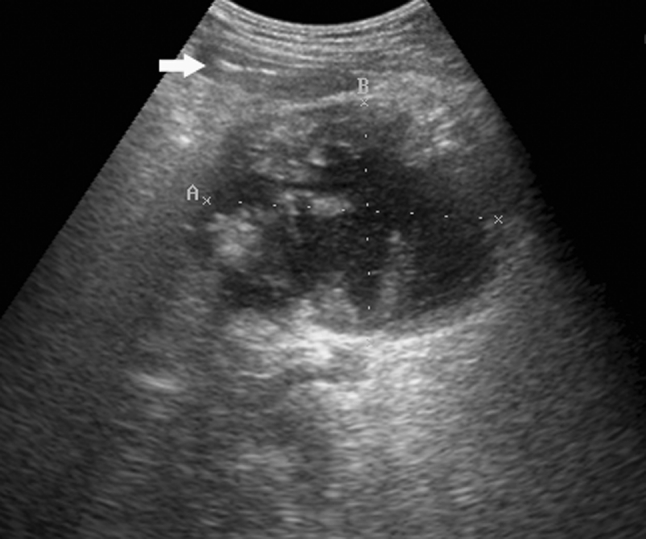
Transverse ultrasound scan demonstrating a fluid–solid collection filled with the necrotic debris (walled-off peripancreatic necrosis), which extends along the descending colon (*arrow*)

**Fig. 2:**
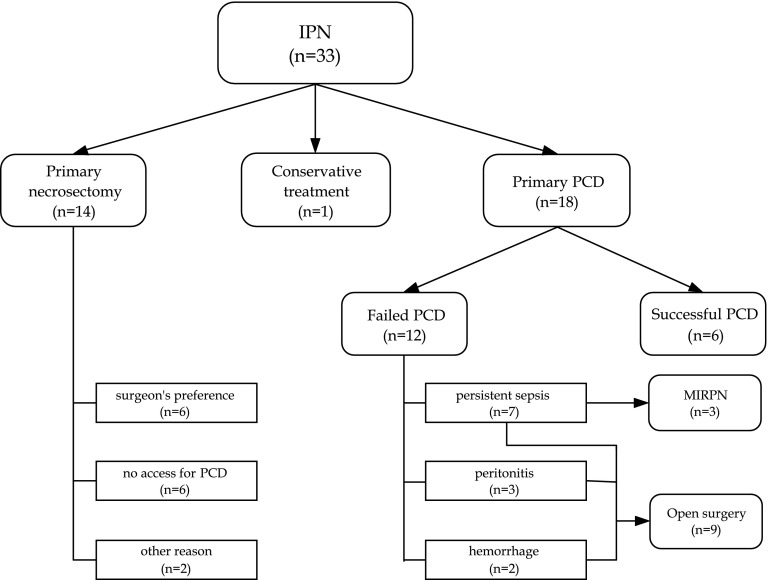
Flow chart demonstrating the process of patient selection and therapeutic management for infected pancreatic necrosis.* IPN* infected pancreatic necrosis,* PCD* percutaneous catheter drainage,* MIRPN* minimally invasive retroperitoneal pancreatic necrosectomy

The indication for PCD in this series was a deteriorating clinical condition of the patient with persistent fever, increasing C-reactive protein level (CRP) and leucocytosis or presence of peripancreatic gas on CT scans. The site and technique of percutaneous catheter drainage was chosen based on the location, size and extent of the peripancreatic collections. The route of access was planned by means of transabdominal ultrasonography, and the free-hand technique was used for placement of the catheters into the liquid area of the necrosis in most cases. Our preferred approach was retroperitoneal through the left lumbar access with the path located between the left kidney and the descending colon (Fig. 1). When this route was not feasible, the drainage was alternatively performed using the anterior transperitoneal access through the gastrocolic ligament or other transperitoneal access in selected cases. The collections located in the right perirenal space were approached retroperitoneally through the right lumbar access between the kidney and the ascending colon. Tandem trocar technique was used for placement of the catheters that were 12F in size or smaller. Larger catheters were inserted using the Seldinger technique. The catheters of more than 16F in diameter were used preferably for replacement and required prior instrumental dilatation of the access path. One catheter was used for each access site and the drains were left for gravidity drainage and irrigated with saline at least once a day. The catheters were upsized or additional drainage was established in patients with persistent local sepsis. The procedures were performed under local anesthesia, and general anesthesia was used for patient’s comfort in selected cases. After the drainage procedure, antibiotic therapy was modified according to the susceptibility report and continued for at least 7 days. The indication for crossover to surgical debridement was lack of improvement despite of percutaneous drainage with large-bore catheters or complications requiring surgery. Up to 2009, open necrosectomy was performed after failure of percutaneous drainage. Thereafter, minimally invasive retroperitoneal necrosectomy was attempted preferably in patients with percutaneous drainage previously established through the left lumbar access. Our technique of minimally invasive retroperitoneal pancreatic necrosectomy using single-port access has been reported elsewhere [5]. The process of patient selection and therapeutic management for infected pancreatic necrosis in this series is illustrated in Fig. 2.

### Statistical analysis

Statistical analysis was performed using Statistica 10 software (StatSoft Poland). Descriptive statistics were used including mean ± SD, median and range. The Mann–Whitney *U* test was used for continuous data, and the Fisher exact test was used for categorical data analysis. A *p* value of <0.05 was regarded statistically significant.

## Results

The indication for percutaneous catheter drainage of pancreatic necrosis in this series was invariably clinical deterioration of the patient’s condition with increasing inflammatory parameters and persistent fever. The median level of serum CRP before the initial drainage procedure was 251 mg/L (range, 122–501 mg/L), and the median leucocytosis was 14 × 10^9^/L (range, 5.6–45.0 × 10^9^/L). In 2 cases, CT scans revealed the presence of peripancreatic gas. The median interval from the onset of acute pancreatitis to percutaneous catheter drainage was similar in the group successfully treated by PCD (33 days) and in the group of cases of failure of percutaneous drainage (25 days). However, there was a higher proportion of initial interventions performed within the first 4 weeks of disease in the patients who failed PCD than in the group after successful PCD, although statistically not significant. Culture of the specimens taken at the initial drainage procedure grew single microorganisms in the majority of the patients (67 %). *Staphylococcus aureus* was isolated in 7 cases (39 %), *Escherichia coli* in 4 (22 %), *Enterococcus* spp. in 3 (17 %) and *Candida albicans* in 2 (11 %).

Necrotic collections were preferably approached retroperitoneally through the left lumbar access (13 of 18 patients). In all but one of these cases, the collection extended at least down to the lower pole of the left kidney. Six of these patients required additional placement of a catheter using other access (transperitoneal or right retroperitoneal each in three cases) because of a collection that was inaccessible or undrainable through the left lumbar access. The transperitoneal access through the gastrocolic ligament alone was used in 4 patients. In one patient, the collection was accessed transperitoneally in the lower abdomen. The details of PCD and treatment outcomes are shown in Table 2.

**Table 2:** Technical details of percutaneous catheter drainage and outcomes

Variable	Successful PCD (*n* = 6)	Failed PCD (*n* = 12)	*p*
Time to PCD, days, median (range)	33 (27–46)	25 (8–116)	0.29
First PCD within/after 4 weeks of disease, no.	1:5	7:5	0.15
Catheter size, F, median (range)	14 (9–32)	14 (9–28)	0.96
No. of catheters, median (range)	2 (1–3)	1 (1–2)	0.21
Duration of drainage, days, median (range)	53 (13–156)	8.5 (1–53)	0.01
Access route, *n* (%)			
Retroperitoneal^a^/transperitoneal alone	3:3	10:2	0.27
Mortality, *n* (%)	0	3 (17)	NA

*PCD* percutaneous catheter drainage

^a^Three patients required additional transperitoneal access

A median of 1 catheter (range, 1–3) was used per patient, whereas the median size of the catheters was 14F (range, 9–32F), and these did not differ between the study subgroups. Percutaneous catheter drainage resulted in complete resolution of infected pancreatic necrosis in 6 out of 18 patients (33 %). One of these 6 patients required open cholecystectomy for acute gangrenous cholecystitis that was performed through the Kocher incision, but no formal necrosectomy was done at the time of laparotomy. In another case, PCD resulted in resolution of the infection involving the lesser sac, but a small infrarenal collection (20 × 50 mm in size) of residual necrotic debris was evacuated digitally under sonographic guidance using a 2–3-cm incision in the left flank. This collection was predominantly solid, and therefore a typical percutaneous catheter drainage, which requires a substantial liquid contents, was not performed. We did not consider this additional procedure as failure of PCD.

Twelve of 18 patients with infected pancreatic necrosis who were initially managed with PCD required eventually crossover to necrosectomy. The most common reason for surgical debridement in this series was persistent sepsis due to failure to evacuate the solid debris despite of a prolonged percutaneous drainage (*n* = 7). Open necrosectomy was performed in four of these patients. The remaining three patients underwent successful minimally invasive retroperitoneal necrosectomy, and none of them required open surgery. Five patients required conversion to open surgery because of procedure-related complications. In three patients, there was leakage of the necrotic material into the peritoneal cavity resulting in diffuse peritonitis. In two of these cases, the peritoneum was inadvertently injured at the time of initial catheter placement using the lumbar retroperitoneal access. In another case, the leakage occurred when the catheter, which had been placed transperitoneally into the lesser sac, slipped out accidently after a week of effective drainage.

Hemorrhagic complications occurred in two patients. One patient experienced a massive septic bleeding from the splenic artery in the third week of PCD and underwent emergent distal pancreatectomy and splenectomy. In another patient, there was bleeding from the gastrocolic ligament upon upsizing of the catheter through the anterior transperitoneal access what required conversion to open technique.

Worsening of organ function was observed early after PCD in only one patient. New-onset organ failure did not occur in any of the patients who underwent minimally invasive retroperitoneal pancreatic necrosectomy. In contrast, 4 of the 10 patients, who had no organ failure preoperatively, developed new-onset organ dysfunction soon after open necrosectomy.

One of the six patients who recovered on PCD alone developed a pancreaticocutaneous fistula that closed spontaneously within 2 months.

The size of the necrotic collection in the patients who were successfully treated with percutaneous catheter drainage alone decreased by a median of 76 % (range, 45–83 %) within a median of 16 days after PCD (range, 7–28 days). In comparison, the size of the collection in the patients who failed percutaneous drainage and required necrosectomy for persistent sepsis decreased only by a median of 16 % (range, 12–19 %) within a median of 10 days after PCD (range, 8–12 days).

The median duration of percutaneous drainage in the patients who were treated successfully with this technique was significantly longer than in patients who failed PCD, 53 versus 8 days. However, a high proportion of patients in the latter group experienced technical complications at the initial drainage procedure, which required open surgery already on the same day or persistent sepsis prompted surgical intervention.

The overall mortality in this series was 17 % (3 of 18 patients). Two patients succumbed to multiorgan failure and uncontrolled sepsis. One patient died of intracerebral hemorrhage, while recovering from acute pancreatitis. All of these patients underwent open necrosectomy after failed percutaneous drainage. Overall, minimally invasive treatment of infected pancreatic necrosis in this series was successful in 9 out of 18 patients (50 %); 6 patients recovered on percutaneous catheter drainage alone (33 %), and 3 patients after conversion to minimally invasive retroperitoneal necrosectomy (17 %).

## Discussion

The natural course of pancreatic necrosis is associated with gradual liquefaction of the solid debris forming a collection of liquefied necrosis that can finally be absorbed. This process may anytime become complicated by superinfection of the necrotic tissues what usually requires surgical or radiological intervention. Open necrosectomy is still considered the “gold standard” treatment in infected pancreatic necrosis, although it carries, even nowadays, a high mortality rate and significant morbidity [6–8]. Necrosectomy should be performed as late as possible after the onset of acute pancreatitis to allow maximal demarcation and liquefaction of the devitalized tissues [2]. Unsatisfactory results of surgical treatment have prompted an introduction of minimally invasive techniques for pancreatic debridement. Freeny et al. [9] were first to use the technique of percutaneous catheter drainage for the treatment of IPN. Recently, other minimally invasive methods of pancreatic necrosectomy have also been developed including endoscopic [10] and laparoscopic [11] approaches to the necrosis.

The success rate of percutaneous catheter drainage in infected pancreatic necrosis is relatively varied and ranges from 0 to 78 % [9, 12–15]. Van Baal et al. [16] reported a meta-analysis of PCD used as primary treatment for necrotizing pancreatitis, which included 384 patients from 11 studies. Surgical necrosectomy could be avoided in 56 % of the patients and the overall mortality rate was 17 %. However, infected necrosis was confirmed in only 71 % of the patients. In most of the reviewed studies, percutaneous drainage was performed under CT guidance. To the best of our knowledge, only 3 cohort studies using ultrasound-guided percutaneous catheter drainage of infected pancreatic necrosis has been reported in the literature [3, 4, 17], and other published case series used ultrasound-guided PCD inconsistently or only in selected cases. Delattre et al. [3] used percutaneous drainage in 42 patients with infected pancreatic necrosis. Infection resolved in 16 % of the patients, and there was a mortality rate of 17 % in this series. In comparison, Navalho et al. [4] performed ultrasound-guided percutaneous drainage of infected peripancreatic fluid collections in 30 patients, including 21 patients with necrosis. Sixty three percent of the patients were cured by PCD alone with a comparable mortality. Recently, Zerem et al. [17] published the largest cohort of ultrasound-guided PCD in patients with infected pancreatic necrosis. In their series, 58 of 69 patients (84 %) underwent successful percutaneous drainage. Eleven patients required conversion to open surgery (16 %), and the mortality rate was only 8.7 %. Such excellent results might be, at least partially, attributed to a meticulous technique used by a dedicated and experienced team to promote liquefaction and fragmentation of the solid debris including vigorous catheter irrigation and frequent drain manipulations.

Effective percutaneous drainage requires frequent catheter upsizing and exchange. Additional procedures demand serial CT examinations. In a series reported by Bruennler et al. [18], the patients treated with PCD received a median of 6 contrast-enhanced CTs (range, 1–23). Such doses of radiation within a short period of time may obviously prove deleterious. Ultrasound-guided PCD is a technique without radiation hazards and has the advantage of real-time imaging, but is more operator-dependant and provides worse visualization of the retroperitoneal space, especially in obese patients. In our series, some necrotic collections with abundant gas were difficult to differentiate from the bowel loops and PCD was not attempted. In such situation, the CT-guided technique seems to be a better alternative. The CT-guided technique often employs multiple catheters inserted through the separate access paths. In contrast, it is seldom possible to find more than one or two optimal sites to access a single peripancreatic collection using transabdominal ultrasonography because of the intervening bowel loops.

Modern management of necrotizing pancreatitis involves the so-called “step-up” approach. PCD is usually used as primary treatment and often regarded as a temporizing method to control sepsis and delay operation. The next step is minimally invasive necrosectomy or traditional open necrosectomy. However, this temporary management with PCD frequently proves to be the only treatment necessary. In a series reported by van Santvoort et al. [14] percutaneous catheter drainage was the definitive treatment in approximately a third of the patients with infected necrosis. Similarly, in our series 33 % of patients required only percutaneous drainage and additional 17 % of patients recovered after minimally invasive necrosectomy. In comparison, Zerem et al. [17] reported an outstanding success rate of 84 % in patients with infected necrotizing pancreatitis treated over 20 years.

There are no definitive criteria that allow prediction of which patients with infected pancreatic necrosis are likely to benefit from percutaneous drainage and which patients should be offered early surgical intervention. Horvath et al. [19] found out that a reduction in collection size of 75 % at 10–14 days after PCD predicted the success of percutaneous drainage with 100 % accuracy. In our series, PCD was successful when collection size decreased by a median of 76 %, whereas a negligible reduction in collection size (12–19 %) predicted failure of percutaneous drainage. Moreover, percutaneous catheter drainage seems to be best suited for patients with liquefied necrosis. Similar to open necrosectomy, this procedure should be delayed as long as possible to allow adequate liquefaction of the necrotic debris. In our series, five of the six patients who were successfully treated by PCD underwent the initial drainage procedure for walled-off necrosis after the fourth week of the disease. In contrast, only one patient, who had the first percutaneous intervention for acute necrotic collection, did not require subsequent necrosectomy.

The mortality rate in our series was comparable to other series [3, 4]. Nevertheless, death of a patient after a failed PCD always raises a question whether percutaneous drainage delayed the appropriate treatment, which is open necrosectomy in many cases, and the decision to intervene operatively was taken too late. There were 3 fatal cases in our series. We think that none of these deaths might be attributed to a delayed decision about open necrosectomy. One patient died of intracerebral hemorrhage whereas intra-abdominal infection was controlled and he was already recovering from acute pancreatitis. The second patient had only 2 days of percutaneous drainage before he underwent open necrosectomy. The third patient had percutaneous drainage for 10 days and his condition improved temporarily, but he required surgical debridement eventually for persistent sepsis. On exploration, a duodenal fistula due to focal necrosis was found in the second portion of the duodenum, and such a complication of acute pancreatitis is notorious for its poor prognosis.

**Fig. 3:**
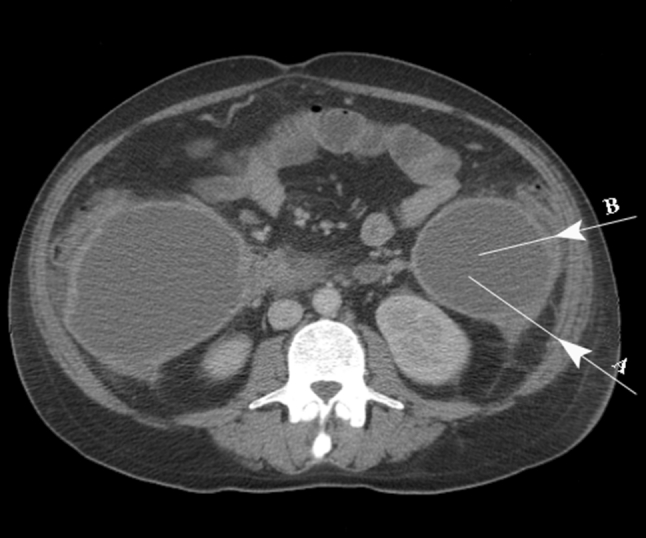
Computed tomography image indicating the appropriate (*arrow A*) and incorrect (*arrow B*) direction for placement of the drainage catheter. Choosing the latter route may result in traversing the peritoneal recessus and leakage of the necrotic material into the peritoneal cavity

Morbidity of percutaneous catheter drainage is varied and ranges between 3 and 60 % [13, 18, 20]. The procedure-related complications are rare, and self-limiting bleedings occur most frequently. In this series, the most common procedure-related complication was diffuse peritonitis due to soiling of the peritoneal cavity with the necrotic debris. On two occasions, this complication resulted from traversing of the peritoneal cavity using the left retroperitoneal access and it was the reason for early failure of PCD. Potential intraperitoneal leakages along the catheter placed through this access seldom seal sufficiently because of poor tissue apposition and lack of the omentum in this region. In addition, the particulate debris clog the catheters and further promote intraperitoneal leakage. These complications occurred early in our experience with this technique and could have probably been avoided using the access path situated more dorsally, which avoids the peritoneum. Although the principal rule in ultrasound-guided drainage procedures is to use the shortest route to collections, this rationale should be cautiously used in patients with infected pancreatic necrosis because the drainage path may pass through the peritoneal recess in the paracolic gutters when the retroperitoneal approach is chosen. In such a situation, inadvertent injury of the peritoneum may lead to reflux of necrotic contents into the peritoneal cavity and cause peritonitis. Therefore, the access site in case of retroperitoneal approach should be located close to the posterior axillary line in order to avoid entering the peritoneal cavity. The appropriate direction for placement of a percutaneous drainage catheter through the lumbar access is illustrated in Fig. 3. In one case, diffuse peritonitis occurred surprisingly also after an accidental dislodgement of the catheter inserted through the anterior transperitoneal approach. In general, the anterior transperitoneal access through the gastrocolic ligament can be done safely because local inflammation affecting the tissues overlying the pancreas and the omentum cause their diffuse and tight adherence to the abdominal wall, which easily seals any leakages.

Hemorrhagic complications after percutaneous drainage are usually self-limiting. Nevertheless, two cases of massive hemorrhage due to injury of the splenic artery have been reported in the literature and both proved fatal [21, 22]. In our series, the case of hemorrhage from the splenic artery resulted from local sepsis and was not related to the procedure itself. Another bleeding complication in our series occurred upon upsizing of the catheter through the gastrocolic ligament. The drawback of this access is a high risk of hemorrhage because of congested vasculature within the gastrocolic ligament in patients with pancreatitis.

The left retroperitoneal route offers the optimal access to the pancreatic necrosis. In our opinion, the prerequisite for a safe retroperitoneal catheter drainage under sonographic guidance is extension of the peripancreatic collections at least down to the lower pole of the kidney. Moreover, this approach is ideally suited for subsequent minimally invasive pancreatic necrosectomy, should percutaneous drainage fail. This technique of necrosectomy allows fairly straightforward access to the necrosis extending even up to the lesser sac. We used this technique in 3 of our patients after failed PCD and all of them avoided open surgery, although 1 or 2 sessions of retroperitoneoscopic necrosectomy were necessary.

This study has some limitations. First, this is a retrospective study and the patients group is relatively small and a substantial number of patients are referrals what precluded evaluation of severity of acute pancreatitis. Second, the study represents the evolution of a PCD-based management of infected pancreatic necrosis in our institution and reflects the learning-curve stage. Therefore, a lower rate of procedure-related complications might be expected with adequate experience and appropriate technique. Nevertheless, US-guided percutaneous catheter drainage offers an interesting alternative to the CT-guided technique in selected cases of IPN with comparable morbidity and mortality. However, a dedicated team is required to successfully manage this challenging group of patients. Further prospective multicenter studies are necessary to confirm the validity of this technique and to define better the factors that influence the efficacy of percutaneous drainage used in infected pancreatic necrosis.

## Conclusions

Ultrasound-guided percutaneous catheter drainage in infected pancreatic necrosis is a technique with acceptably low morbidity and mortality, which may be the definitive treatment or a bridge management to necrosectomy. A negligible decrease in size of the necrotic collection shortly after PCD portends failure of percutaneous drainage.
